# On the BACB’s Ethics Requirements: A Response to Rosenberg and Schwartz (2019)

**DOI:** 10.1007/s40617-020-00463-6

**Published:** 2020-06-12

**Authors:** Tyra P. Sellers, James E. Carr, Melissa R. Nosik

**Affiliations:** Behavior Analyst Certification Board, 7950 Shaffer Parkway, Littleton, CO 80127 USA

**Keywords:** BACB, Behavior analysts, Ethics, Ethics code

## Abstract

Rosenberg and Schwartz (*Behavior Analysis in Practice*, *12*, 473–482, 2019) criticize a number of aspects of the Behavior Analyst Certification Board’s *Professional and Ethical Compliance Code for Behavior Analysts* and propose, as an alternative, a decision-making process for evaluating the ethicality of behavior under a particular set of circumstances. We respond to the authors’ main criticisms and discuss the broader professional and legal context of any profession’s ethics code and enforcement activity.

Rosenberg and Schwartz (2019) identify two key factors that have increased the value of scholarship about ethics in behavior-analytic practice. The first is that the majority of practicing behavior analysts currently provide services to vulnerable client populations (e.g., children with autism spectrum disorder). The second is the robust demand for behavior-analytic practitioners (Behavior Analyst Certification Board® [BACB®], 2020), which has led to a significant increase in the number of Board Certified Behavior Analysts® (BCBAs®) in recent years (BACB, n.d.). Given this context, Rosenberg and Schwartz highlight the need for additional resources on ethics. We wholeheartedly agree with this conclusion. In this commentary, we highlight and respond to the main elements of the Rosenberg and Schwartz article, including their criticisms of BACB ethics standards. Our responses are contextualized within the established and legally based practices of major professions.

Rosenberg and Schwartz’s (2019) primary contribution is the development of a decision-making process for evaluating the ethicality of behavior under a particular set of circumstances. This kind of decision-making algorithm has increasingly been used in recent years as an approach for guiding practitioners through a variety of practice-related decisions (e.g., Geiger, Carr, & LeBlanc, 2010; LeBlanc, Raetz, Sellers, & Carr, 2016; Virués-Ortega et al., 2014). Rosenberg and Schwartz’s process includes thoughtful and practical questions about relevant contextual variables, which are likely to be helpful prompts for behaving ethically. The authors propose their decision-making process as an alternative to the *Professional and Ethical Compliance Code for Behavior Analysts* (BACB, 2014; hereafter referred to as “the Code”) based on four major criticisms of the Code: (a) its seeming insensitivity to culture and context, (b) its rule-based nature, (c) its inclusion of apparently conflicting Code elements, and (d) the intent behind and the language used to describe the Code itself. We address each of these four main criticisms in this order.

Rosenberg and Schwartz (2019) make the important point that some ethical dilemmas cannot be addressed in a dichotomous manner (i.e., behavior is either ethical or unethical); instead, these dilemmas require careful consideration of the relevant contextual and cultural variables and the potential outcomes of various actions. For example, the authors address the Code’s elements related to multiple relationships and gifts (1.06). The authors refer to element 1.06(a)—“Due to the potentially harmful effects of multiple relationships, behavior analysts avoid multiple relationships” (BACB, 2014, p. 5)—to illustrate situations in which strictly following this element might lead to suboptimal client outcomes. The authors use their decision-making process to identify the potential risks of initiating a multiple relationship and either avoiding it or managing it by developing actions to minimize these risks. We agree with this approach because of the subjectivity and interpretation variations that can occur when using a code of ethics. In fact, elements 1.06(b) and (c) were specifically included in the Code to encourage this type of analysis. These elements are as follows:

- (b) Behavior analysts must always be sensitive to the potentially harmful effects of multiple relationships. If behavior analysts find that, due to unforeseen factors, a multiple relationship has arisen, they seek to resolve it.
- (c) Behavior analysts recognize and inform clients and supervisees about the potential harmful effects of multiple relationships. (BACB, 2014, p. 5)

The critical word “avoid” in element 1.06(a) was specifically included in the Code to help prompt a contextual analysis that considers potentially influential variables, including culture, diversity, language, and other status-based factors (e.g., education level, socioeconomic status). A more prescriptive phrase, such as “do not engage in” could have been used, which would have implied a contextually inflexible course of action. Furthermore, elements 1.06(b) and (c) are clearly directed at situations in which a behavior analyst is in a multiple relationship, and the elements indicate that the appropriate course of action is to communicate with the individuals involved before working toward a resolution. The May 2015 *BACB Newsletter* addresses both gifts and multiple relationships by providing practitioners with contextual elaborations on these specific Code elements, such as “Context is always considered by a BACB Review Committee; if professional judgment can be described that demonstrates that rejecting the gift would have proven more harmful than otherwise, then a violation might not have occurred” (BACB, 2015, p. 2). Said another way, a professional code of ethics could never outline all of the relevant and ever-changing contextual variables that should inform the application of ethical principles; therefore, it is up to the practitioner to actively and thoughtfully consider specific variables in applying professional judgment to potential or actual ethical dilemmas. For recommendations on strategies for considering culture and diversity in behavior analysis, readers are directed to the helpful article by Fong, Ficklin, and Lee (2017).

Rosenberg and Schwartz (2019) characterize certain Code elements as being “in conflict” (i.e., one could not simultaneously meet both elements), but such conflicts are commonplace within professional ethics codes. A cursory review of ethics codes published by the American Medical Association, the American Psychological Association, the American Speech-Language-Hearing Association, the National Association of Social Workers, and the American Occupational Therapy Association reveals that all include potentially conflicting code elements (e.g., multiple relationships, discrimination, bartering for services). The authors describe Code elements 1.05(f) (refraining from working if services are compromised by personal circumstances) and 1.04(c) (following through on work commitments) as being in conflict in a situation wherein a practitioner’s divorce might negatively impact his or her ability to complete work tasks. However, an alternative analysis of this apparent conflict might be that Code elements can interact in a complementary and supporting manner. Taking this alternative approach to the authors’ example, one might proceed in the following manner: (a) if applying 1.05(f) led a behavior analyst to determine that a personal event was compromising service delivery, (b) a subsequent application of 1.04(c) ensuring follow-through on work commitments might (c) result in the behavior analyst reaching out to a colleague for assistance with impending tasks. In other words, simply stating that Code elements are in opposition may result in missed opportunities to engage in ethical decision making using the Code elements as guides that would actually neutralize apparent conflicts.

Rosenberg and Schwartz (2019) primarily focus on the role of the Code as a document that influences the behavior of a practitioner, but they do not address the other critical functions of an ethics code. A clear, rule-based ethics code provides a framework for practitioners and consumers to evaluate a practitioner’s behavior to (a) gain an understanding about questionable behavior, (b) help identify more ethical courses of action, and (c) determine when to report potentially unethical behavior. This last function highlights the Code’s role as the foundation of a disciplinary system for addressing practitioner misconduct. Every regulated helping profession has a rule-based ethics code (e.g., psychology, medicine, occupational therapy, speech-language pathology, social work) that is enforced through a variety of disciplinary mechanisms^1^. Such enforcement can occur at the national level, typically by certification boards (e.g., the BACB, the National Board for Certification of Occupational Therapists, the American Speech-Language-Hearing Association), and at the state level by licensure boards. These critical systems help protect consumers of services by addressing the unethical behavior of practitioners.

Rosenberg and Schwartz (2019) focus exclusively on the Code and its influence on practitioner behavior in isolation, rather than within the broader context of the BACB’s activities. The system that the BACB operates for addressing alleged Code violations affords the subject of a complaint due process under the law, including an opportunity for the subject to review the allegation and supporting documentation, respond and provide contextual information about the incident, and receive a peer review of the case and an opportunity to appeal disciplinary decisions. In addition, the Code is not the only mechanism for influencing practitioner behavior, but it is supported by certification eligibility requirements (e.g., a 45-hr stand-alone course on ethics for BCBA applicants) and maintenance requirements (e.g., 4 hr of continuing education in ethics every 2 years for BCBAs).

It is worth noting that there are many philosophical paradigms that can inform professional codes of ethics, as well as a practitioner’s application of ethics principles and Code elements to a given dilemma. A thoroughgoing treatment of these paradigms is beyond the scope of this article; therefore, we refer readers to Brodhead, Cox, and Quigley (2018) for a fuller treatment of the topic. Rosenberg and Schwartz are not wrong in their assumption that the Code is informed by a deontological, or rule-based, approach to ethics. However, we assert that the rule-based underpinnings of the Code elements do not preclude individuals from gaining insight from other paradigms of applied ethics (e.g., utilitarianism or virtue ethics) when interpreting and applying Code elements to a specific situation. As stated previously, Rosenberg and Schwartz’s framework for guiding ethicality appears quite useful for assisting practitioners in addressing a particular ethical dilemma but could not serve as the basis of a legally defensible ethics enforcement system. Although it is possible that disciplinary mechanisms could exist without a rule-based code, we have yet to identify such a system.

Finally, Rosenberg and Schwartz (2019) characterize the shift from using “guidelines” to “compliance” in the titles of the BACB’s ethics codes as representing a meaningful change in its function. This characterization is incorrect. As the authors indicate, the original version of the *Guidelines for Responsible Conduct for Behavior Analysts* (BACB, 2001) was not enforced by the BACB. However, this lack of enforcement did not result from a determination that practitioners only needed guidance. It was instead a reflection of the nascent state of the BACB and its limited resources (e.g., at the time, the BACB had a single unpaid employee on staff). That said, the *Professional Disciplinary Standards* did provide a mechanism for addressing substantial practitioner misconduct through standard 6, “Gross or repeated negligence, incompetence or malpractice in professional work” (BACB, 1999, p. 1). A revision of these disciplinary standards enacted in 2010 did explicitly link the entirety of a revised version of the *Guidelines for Responsible Conduct for Behavior Analysts* (BACB, 2010) to the enforceable *Professional Disciplinary Standards* (BACB, 2010) by including “misconduct” in standard 6 (“gross or repeated negligence, incompetence, misconduct, or malpractice in professional work,” p. 1). In 2014, the BACB introduced the Code, which combined the earlier conduct guidelines and disciplinary standards into one enforceable document. All of the BACB’s ethics codes have been rule based since 2001 and enforceable since 2010 (with broader malpractice being enforceable as early as 1999). Thus, although Rosenberg and Schwartz place much emphasis on the semantic shift from “guidelines” to “compliance” in the ethics codes’ titles, this change in terminology does not, in fact, convey a shift in the BACB’s approach to ethics or disciplinary enforcement.

In conclusion, we are heartened by the many contributions by authors who are newly writing about the state of our profession, such as the algorithm for supporting ethics-related decision making by Rosenberg and Schwartz (2019). It is important to bear in mind that a profession’s infrastructure (e.g., educational programs, professional association activity, licensure) is greatly impacted by a nation’s laws. For example, professional association and certification/licensure activity in the United States is heavily influenced by antitrust legislation, Federal Trade Commission opinions, and Supreme Court rulings. Thus, although behavior analysts have devised better methods of solving problems in a variety of areas, there are limits to the implementation of new behavior-analytic approaches in areas that have established and inflexible processes, which should be acknowledged. BACB staff are diligent about researching the practices of other professions and legal limitations, and providing that information to subject matter experts to consider in their determinations about BACB certification standards. We encourage authors who write about our profession and its various requirements (e.g., credentialing, public policies, examination administration, ethics) to properly contextualize them by referencing the established, and sometimes required, practices of related professions.

## Footnotes

1 Comments in this article are provided in the context of U.S.-based professional regulation.
